# Basic Principles of Rotational Thromboelastometry (ROTEM^®^) and the Role of ROTEM—Guided Fibrinogen Replacement Therapy in the Management of Coagulopathies

**DOI:** 10.3390/diagnostics13203219

**Published:** 2023-10-16

**Authors:** Miroslava Drotarova, Jana Zolkova, Kristina Maria Belakova, Monika Brunclikova, Ingrid Skornova, Jan Stasko, Tomas Simurda

**Affiliations:** National Centre of Hemostasis and Thrombosis, Department of Hematology and Transfusiology, Jessenius Faculty of Medicine in Martin, University Hospital in Martin, Comenius University in Bratislava, 036 01 Martin, Slovakia; miroslava.sterankova11@gmail.com (M.D.); jana.zolkova@gmail.com (J.Z.); kristinabelakova@gmail.com (K.M.B.); simkovamonika@gmail.com (M.B.); inkaskornova@gmail.com (I.S.); jan.stasko@uniba.sk (J.S.)

**Keywords:** rotational thromboelastometry, management, fibrinogen disorders, fibrinogen replacement therapy

## Abstract

Rotational thromboelastometry (ROTEM) is a viscoelastic method, which provides a graphical and numerical representation of induced hemostasis in whole blood samples. Its ability to quickly assess the state of hemostasis is used in the management of bleeding from a variety of causes. The separate activation of particular parts of hemocoagulation in INTEM, EXTEM, and FIBTEM tests allows for a more comprehensive and faster evaluation of the missing component of hemostasis followed by targeted therapy. One of the most common cause of coagulopathy is trauma-induced coagulopathy. Fibrinogen replacement therapy by ROTEM allows for the use of a standard dosage of fibrinogen, which has been shown to be successful in preventing dilutional coagulopathy following colloid and crystalloid replacement and excessive amount of allogeneic blood transfusions. The best reflection of fibrinogen activity is observed in the FIBTEM assay, where fibrinogen replacement therapy is recommended at an MCF (maximum clot firmness) of FIBTEM < 10 mm and FIBTEM A10 < 7 mm. ROTEM also plays an important role in the diagnostic and management of inherited fibrinogen disorders. These can be manifested by bleeding complications, where changes in the MCF parameter are the most useful tool for assessing the effectiveness of fibrinogen replacement therapy. ROTEM-guided bleeding management algorithms effectively reduce the number of transfusions, healthcare costs, and complications, leading to the improvement of patient safety and overall health.

## 1. Introduction

Rotational thromboelastometry (ROTEM) is a point-of-care testing devices, which means that tests can be performed at the bedside. Test results are delivered quickly, enabling rapid treatment modification [1]. Increasingly, ROTEM analysis is being incorporated into the diagnostic algorithm and the treatment of bleeding in high-risk patients, such as those undergoing cardiac surgery or suffering from extensive trauma. This system also plays an important role in the management of peri- and postpartum bleeding, in the diagnosis of inherited and acquired bleeding disorders, or in complex procedures, such as liver transplantation. According to the latest findings, ROTEM, as a fast and reliable diagnostic tool, is an essential component of blood management since it can significantly reduce the number of allogeneic transfusions and increase patient safety [2].

## 2. The Measuring Principle of ROTEM

The ROTEM device measures changes in viscoelastic properties in blood during clot formation in a small sample of citrated or heparinized blood (300–340 μL) after the addition of clotting factors. A blood sample is heated to 37 °C in a stationary disposable cup, which is subjected to the constant rotational force of an oscillating pin. The oscillating axis has an attached mirror to which a light beam is directed. As the clot forms around the pin, pin oscillation is increasingly restricted, and changes in light reflectance are captured by a photodetector [2,3,4].

**Figure 1:**
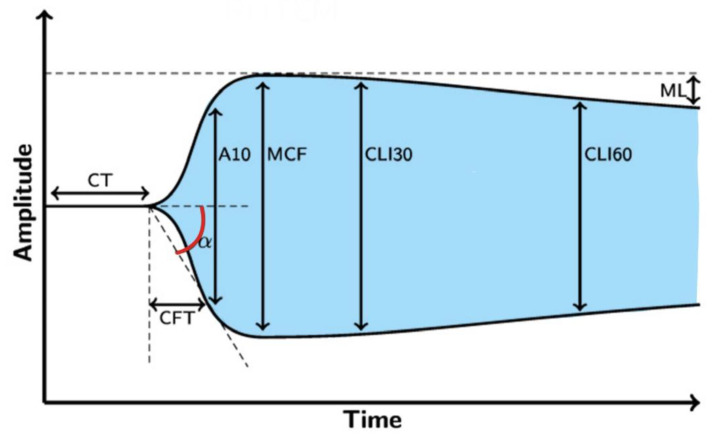
The resulting curve of rotational thromboelastometry. Legend: CT—clotting time (s), CFT—clot formation time (s), α—angle of clot polymerization rate (°), A10—clot strength value in time of 10 min from CT (mm), MCF—maximum clot firmness (mm), CLI30—lysis index 30 min after clotting time (%), CLI60—lysis index 60 min after clotting time (%), ML—maximum clot lysis (%).

Thus, based on the emerging thromboelastometry curve (Figure 1), we can visually evaluate coagulation from the formation of the clot through its promotion and stabilization of its dissolution in the process of fibrinolysis. At the end of the examination, based on computer analysis, we can obtain an extensive set of kinetic parameters that arise during the sophisticated process of blood clot formation.

The advantage of this POC test is the possibility of separating the activation of the intrinsic coagulation cascade (by kaolin and ellagic acid) and the extrinsic coagulation cascade (by tissue factor), which helps evaluate the functionality of the particular components of coagulation and thus allows for targeted therapeutic interventions in terms of the missing factor substitution. At the same time, after the addition of a platelet inhibitor, independent of platelet influence, it is possible to monitor the influence of fibrinogen on the final clot strength [5].

The ROTEM system allows for four independent measurements that can be performed independently of each other in separate chambers, giving a comprehensive view of patient’s coagulation. 

### 2.1. INTEM Assay

Negatively charged surfaces (kaolin or ellagic acid) activate the intrinsic pathway of the coagulation cascade in the INTEM tests, thus providing information similar to that provided by the activated partial thromboplastin time (aPTT) examination, especially the clotting time (CT) parameter.

### 2.2. EXTEM Assay

By using tissue factor as a reagent, it is possible to assess the extrinsic pathway of the coagulation cascade, with clotting occurring faster than in the intrinsic coagulation pathway assay, similar to the living organism. The resulting parameters of the EXTEM assay correspond to the prothrombin time (PT) in basic coagulation. Similar to the INTEM and APTEM assays, clot strength is mainly influenced by fibrinogen and platelets [6]. Pathological values of particular parameters reflect a factor deficiency of the extrinsic coagulation pathway, as well as the possible influence of vitamin K antagonists or direct-acting oral anticoagulants (DOACs).

### 2.3. FIBTEM Assay

The addition of cytochalasin, which has an inhibitory effect on myctofilaments in platelets, prevents platelet-mediated clot retraction. Thus, the FIBTEM test best reflects fibrinogen activity. FIBTEM also correlates very well with fibrinogen activity determined by the Clauss assay [7]. The Clauss fibrinogen assay is the most often used laboratory method to measure plasma fibrinogen levels. The main advantage of the FIBTEM assay is that it provides information on fibrinogen activity within minutes, whereas the Clauss test requires 30–60 min before the result is available [7]. There is an evidence that early FIBTEM assay parameters predict the final strength of the blood clot. The FIBTEM assay detects fibrinolysis sooner than assays for the intrinsic and extrinsic pathways of blood coagulation. Therefore, this type of fibrinogen determination should be used in the management of active bleeding in trauma patients to provide early antifibrinolytic therapy [8].

### 2.4. APTEM Assay

The addition of the plasmin inhibitor aprotinin as a reagent is used to assess and confirm whether the decrease in clot amplitude is caused by fibrinolysis. The results are compared with those of the EXTEM assay, with shortened clotting time (CT) and higher maximum clot strength (MCF) in the APETM test indicating fibrinolysis.

### 2.5. HEPTEM Assay

HAPTEM test is an optional used in heparinized patients. Due to the fact that the INTEM assay is particularly sensitive to heparin, leading to a distortion of the results, in the HEPTEM assay, the effect of heparin is inhibited by adding heparinase, and the results are compared to the parameters in the INTEM assay. Thus, the inhibitory effect of heparin on coagulation is proven.

Five possible examinations (INTEM, EXTEM, FIBTEM, APTEM, HEPTEM) together with the necessary reagents are summarized in Table 1.

## 3. Interpretation of Results

In the initial phase of blood clotting, the coagulation factors of both the external and internal coagulation pathways play the most important role. The role of these factors can be observed as the CT value in the INTEM, EXTEM and HEPTEM assays, as hypercoagulability is demonstrated by a shortened CT value, and vice versa, i.e., hypocoagulability or the presence of an anticoagulant determines a prolonged CT value.

In the next phase of the kinetics of clot formation, called amplification, fibrinogen plays a decisive role. The FIBTEM test and the values of CFT (s) and the α angle (°) measures fibrin kinetics during clot formation.

**Figure 2:**
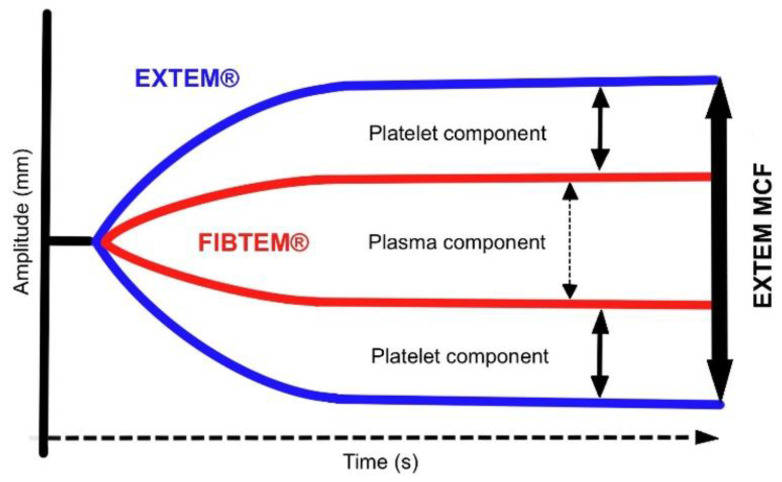
Plasma and platelet component of the result MCF of ROTEM (modified by [10]).

In addition to fibrinogen, platelets also play an irreplaceable role in the process of clot propagation and strengthening. The contribution of platelets and fibrinogen to clot strength and, at the same time, the maximum platelet potential at maximum thrombin stimulation reflects the MCF parameter. In this case, there is the greatest reduction in oscillation, as the plasma and platelet component contribute to the total amplitude size of the MCF in the EXTEM assay (Figure 2).

In the final phase of coagulation, the fibrinolysis phase, the function of fibrinolytic enzymes and inhibitors as well as the role of FXIII is reflected in the ratio between the CLI30 value and the CLI 60. This is defined as a simple percent reduction in amplitude compared to the MCF and the ML value (%). Hyperfibrinolysis is confirmed by increased clot lysis, which begins after 30 min from the beginning of clot formation.

The reference range may vary slightly depending on the relevant control group, namely, the site, the patient age, and the sampling technique, in pregnant women and in its transport as well as other factors during the pre-analytical phase [2]. The reference values for the ROTEM^®^ delta device in adult patients are listed in Table 2.

## 4. The Importance of ROTEM Examination in the Management of Acquired Coagulopathies

**Figure 3:**
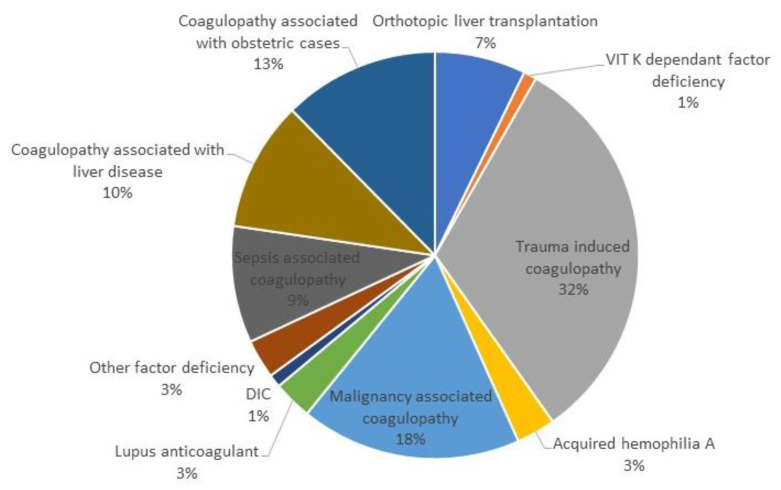
The most common causes of acquired coagulopathy (modified by [12]) Legend: VIT K—vitamin K, DIC—disseminated intravascular coagulopathy.

According to several studies, in approximately 25% to 30% of patients who had suffered trauma, coagulopathy is present [11]. It is also the most common cause of coagulopathy overall, followed by coagulopathy associated with malignancy. Other causes of coagulopathy are listed in Figure 3.

In the case of massive blood loss, the administration of a colloid and crystalloid solutions is an important rescue step. Dilutional coagulopathy and other hemostasis impairments may have contributed to this finding. Severe bleeding ultimately increases mortality, which is probably not only the result of increased perfusion pressure after fluid therapy, but also a consequence of the dilution of clotting factors and the harmful effect of colloids on the coagulation system as well as the disruption of the reticular fibrin mesh and the reduction in clot quality after the administration of gelatin solutions. It seems that fibrinogen plays a key role in massive blood loss, while fibrinogen deficiency is clinically more important than loss of other clotting factors or platelets [13,14].

In Central Europe, fibrinogen concentrate is available for the immediate and efficient treatment of fibrinogen deficiency caused by dilutional and consumption coagulopathy. Plasma-based coagulation screening tests as aPTT and PT are inappropriate for monitoring or guiding transfusion therapy, since they only provide information about the initiation phase of clot formation [15]. Precisely for this reason, the targeted administration of fibrinogen as well as prothrombin complex of clotting factors using viscoelastic methods has been shown to be effective in trauma patients.

Based on the revised Trauma and Injury Severity Score (TRISS) or RISC (fibrinogen concentrate administered with FIBTEM MCF < 10 mm), patients treated according to the ROTEM-controlled fibrinogen concentrate and prothrombin complex concentrate (PCC) administration had a lower mortality rate than predicted [16]. According to a retrospective ROTEM study, Blayney, A. et al., 2022 [17], describes that FIBTEM A5 reliably predicts A10 in trauma patients and, together with MCF [7], saves time in the time-critical resuscitation of trauma patients.

In their survey, Tsantes AG et al., 2022 [18], also demonstrated a high prognostic accuracy of ROTEM parameters for excessive bleeding and increased transfusion requirements in a group of patients undergoing hip fracture surgery. In particular, postoperative FIBTEM MCF ≤ 15 mm had significant sensitivity and specificity for the prognosis of excessive bleeding, and similarly, preoperative FIBTEM MCF ≤ 15 mm had high sensitivity and specificity for the prognosis of increased transfusion requirements. The ROTEM test was a better predictor of transfusion or excessive bleeding, which allows blood banks to ensure adequate blood supply or to develop blood salvaging strategies when treating these patients [19].

ROTEM is an essential examination used in cardiovascular surgery. In this type of surgery, medical doctors have to deal with a 30–45 min lasting time window to perform hemostatic interventions between heparin-reversal by protamine and the end of surgery. During this therapeutic window, the residual heparin effect or protamine overdose is detected by ROTEM examination. The CT_IN_/CT_HEP_ ratio correlates well with the anti-Xa determination of the heparin concentration [20].

Patients undergoing cardiac surgery are at higher risk of postoperative bleeding. Thus, there is an increased need for allogenic blood products and high-dose anticoagulation as well as exposure to cardiopulmonary bypass. Several risk factors are known to be associated with an increased risk of bleeding, transfusion, and reoperation, such as advanced age, preoperative dual antiplatelet therapy, impaired platelet function, preoperative anemia, small body surface area, the female gender, and non-elective surgery [21].

In the case of bleeding after cardiovascular surgery, the fibrinogen substitution is crucial. According to Karkouti et al., 2013 [22], a fibrinogen level <2 g/L, which corresponds to A5_FIB_ < 9 mm, is significantly associated with the need for transfusion of more than 5 units of red blood cells. In the therapeutic algorithm, the cut-off value for fibrinogen supplementation is therefore set to A5_FIB_ < 9 mm [23,24]. The first target is A5_FIB_ ≥ 12 mm, corresponding to a fibrinogen concentration ≥2.5 g/L, and in the case of ongoing bleeding, the second target is A5 _FIB_ ≥ 15 mm, corresponding to a fibrinogen concentration ≥3 g/L [25]. However, it has been found that a fibrinogen substitution higher than an A5_FIB_ value equal to 16 mm would not bring any further improvement in patients without bleeding [26].

According to the European Association for Cardio-Thoracic Surgery (EACTS) and the European Association of Cardiothoracic Anaesthesiology (EACTA) guidelines for patient blood management in adult cardiac surgery conditions, prophylactic preoperative fibrinogen administration to reduce postoperative bleeding and transfusion requirements is not recommended. In patients with a low fibrinogen level, however, fibrinogen substitution may be considered to reduce the requirement for transfusion [21].

There is a growing evidence for the use of ROTEM in patients on extracorporeal membrane oxygenation (ECMO) with a great potential to assess hemorrhagic and thromboembolic risk in these patients. Due to the presence of artificial surfaces in extracorporeal circulation and the possible activation of coagulation, sufficient anticoagulation is required because of the risk of bleeding, too. In their prospective study, Giani M et al., 2021 [27], evaluated ROTEM as a tool to assess hemostasis and anticoagulation levels in ECMO patients. By comparing the results with thromboelastography and conventional coagulation tests, they provided a moderate correlation of the INTEM CT (ROTEM) with standard coagulation tests (aPTT, activated clotting time) but still having a limited amount of information on the issue of patients on ECMO.

The transfusion of all blood components (plasma, platelets, and red blood cells) in fixed high ratios is part of a balanced resuscitation strategy in trauma patients [28,29]. The safety and efficacy of these protocols in a management of non-traumatic bleeding is still unclear [30]. Several previous studies, which focused primarily on patients with acute intraoperative hemorrhaging, have demonstrated that increased plasma- and platelet-to-red-blood-cell (RBC) ratios are not associated with lower mortality. One undeniable reason might be the fact that non-traumatic patients suffering from hemorrhage differs phenotypically and physiologically from trauma patients [31].

**Figure 4:**
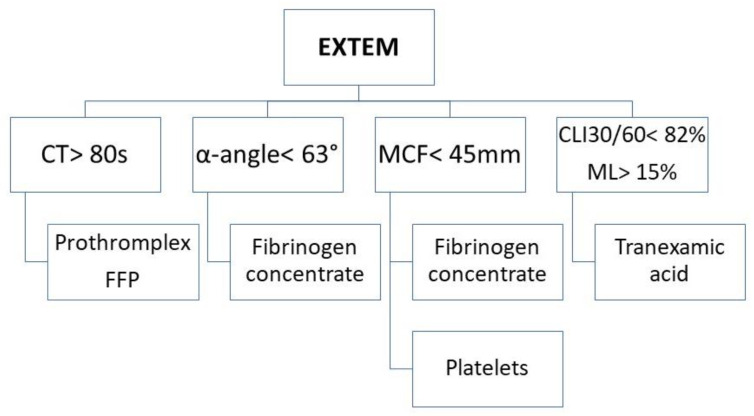
Recommended replacement therapy according to achieved ROTEM value in EXTEM [5,32,33,34]. Legend: FFP—fresh frozen plasma.

**Figure 5:**
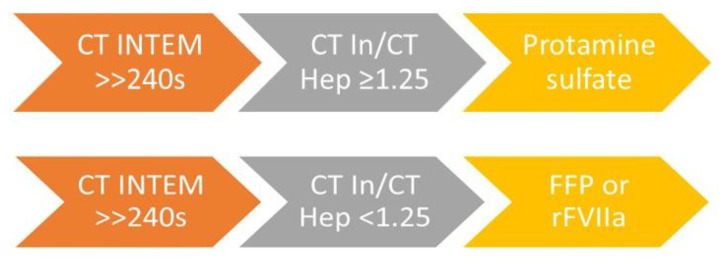
Recommended therapeutic intervention according to achieved ROTEM value in INTEM and HEPTEM [32]. Legend: In—INTEM, Hep—HEPTEM, FFP—fresh frozen plasma, rFVIIa—recombinant activated factor VII.

**Figure 6:**
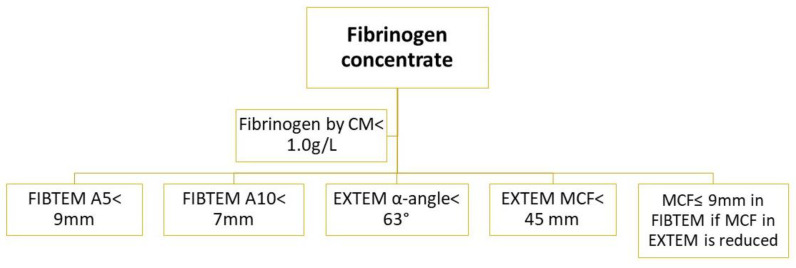
Recommended fibrinogen substitution according to achieved ROTEM value in FIBTEM and EXTEM [5,32,33,34]. Legend: CM—Clauss method.

“Fibrinogen is the first coagulation factor to fall to critically low levels (<1.0 g/L) during major hemorrhage (normal plasma fibrinogen levels range from 2.0 to 4.5 g/L), and current guidelines recommend maintaining the plasma fibrinogen level above 1.5 g/L” [35]. The first evidence of fibrinogen deficiency is often visible in a prolonged CT in EXTEM assay (CT_EX_), but this value can only be adequately interpreted if FIBTEM A5 and FIBTEM A10 assays are performed simultaneously. Consequently, if FIBTEM A5 is to be performed prior to CT_EX_, the test results should be interpreted in a reasonable sequence but not according to their availability (e.g., if CT_EX_ is available prior to FIBTEM A5) [32]. The recommended replacement therapy, according to the achieved ROTEM value in EXTEM, is summarized in Figure 4, and the recommended therapeutic intervention, according to the achieved ROTEM value in INTEM and HEPTEM assays, is summarized in Figure 5.

According to many authors, the cut off value for fibrinogen replacement therapy is when a fibrinogen activity ≤1.0 g/L, according to Clauss method, or MCF ≤ 9 mm in FIBTEM assay if MCF is reduced in EXTEM assay [34]. The recommended fibrinogen substitution according to the achieved ROTEM value in FIBTEM and EXTEM is shown in Figure 6.

Commercial fibrinogen concentrates (RiaSTAP/Haemocomplettan^®^ P (CSL Behring, Marburg, Germany), Fibryga^®^ (Octapharma, Lachen, Switzerland), FibCLOT^®^/CLOTTAFACT^®^ (LFB, Les Ullis, France), Fibrinogen HT^®^ (Benesis, Osaka, Japan), FibroRAAS^®^ (Shangai RAAS, Shanghai, China), and FIB Grifols^®^ (Grifols, Barcelona, Spain) were obtained from pooled human plasma by a cryoprecipitation. In the form of lyophilized powder, it is immediately available for reconstruction using sterile water at room temperature [36,37].

Fibrinogen replacement therapy has a number of advantages: it allows for a standardized fibrinogen dose to be rapidly administered in a small volume, has a very good safety profile, and is virally inactivated during the manufacturing process as a standard. One of the most important advantages for the administration of fibrinogen concentrate (guided by ROTEM) is the possibility of the standardized dosing of fibrinogen in a small volume to infuse fresh frozen plasma (FFP), which usually requires larger volumes to supplement clotting factors, which can be associated with a fluid overload [36]. Fibrinogen concentrate substitution does not require thawing or cross-matching as well [35]. The recommended dosage of fibrinogen for the targeted increase in FIBTEM A10 (A5) amplitude is shown in Table 3.

## 5. The Application of ROTEM in the Management of Bleeding in Patients with Congenital Fibrinogen Disorders

ROTEM-guided substitution therapy also plays an important role in the management of bleeding in patients with inherited fibrinogen disorders (IFD). IFD are rare diseases affecting either the amount of circulating fibrinogen (afibrinogenemia and hypofibrinogenemia), its quality (dysfibrinogenemia), or both (hypodysfibrinogenemia). They are also associated with different clinical phenotypes, including bleeding, and thrombotic or pregnancy complications. Some patients with IFD are also without any clinical manifestation. Most cases of IFD are caused by a causal pathogenic variant in one of the three genes coding for fibrinogen (FGA, FGB, or FGG) [39].

Fibrinogen concentrates were originally approved for the treatment of bleeding episodes in patients with these fibrinogen disorders with an elimination half-life of approximately 80 h. Currently, there are many reasons to consider fibrinogen concentrates as a supplementary therapy for the treatment of coagulopathy [40,41].

**Figure 7:**
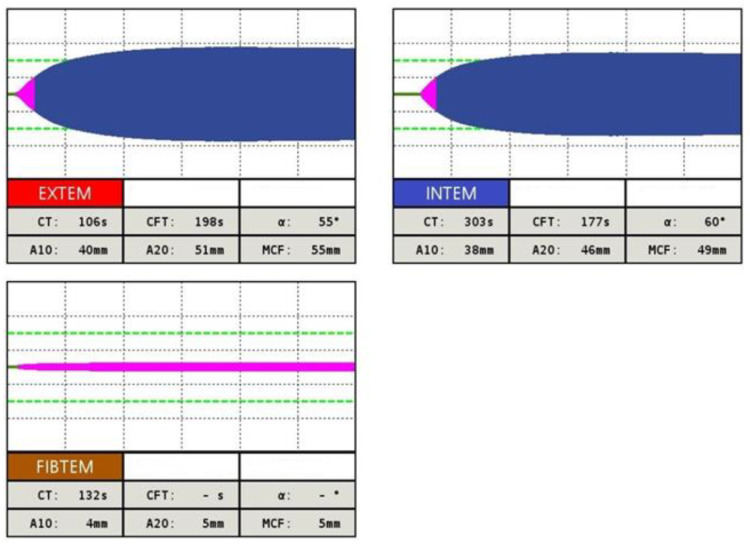
Example of INTEM, EXTEM and FIBTEM assay in patient with inherited dysfibrinogenemia. Thromboelastometric curve in EXTEM test shows prolonged CT and CFT, parameter A10 is shortened. In INTEM test parallel CT and CFT parameters are prolonged, A10, A20 and MCF parameters are shortened. In FIBTEM test a significant decrease in A10, A20 and FIBTEM MCF parameters is observed, what testifies for a clot polymerization disorder due to fibrinogen deficiency.

Rotational thromboelastometry is also a useful tool in the diagnosis of IFD, as the Clauss fibrinogen assay is able to determine the fibrinogen activity, although it does not distinguish between qualitative and quantitative defects of fibrinogen [42]. Currently, the FIBTEM assay is sensitive to the detection of a clot polymerization disorder. Thus, the clot firmness MCF parameter is used to predict fibrinogen deficiency and the need for fibrinogen concentrate [43,44]. An example of a ROTEM test in a patient with congenital dysfibrinogenemia with a significant decrease in the FIBTEM A10 and FIBTEM MCF parameters is presented in Figure 7.

The fibrinogen dose can be calculated according to the following formula: Fibrinogen concentrate dose (g) = (target FIBTEM MCF (mm) − actual FIBTEM MCF (mm)) × (body weight (kg)/70) × 0.5 g/mm [7]. The increase in the MCF also serves as a parameter of efficacy after fibrinogen infusion.

**Figure 8:**
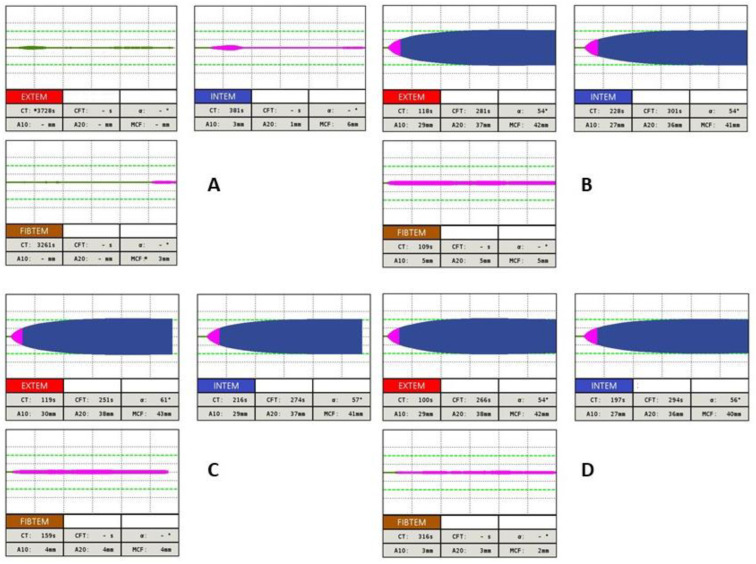
Example of using ROTEM in patient with congenital afibrinogenemia after administration of 23.5 mg/kg fibrinogen concentrate: (**A**) basal values (Fbg 0.54 g/L [ref. 1.80–4.20], PT 74% [ref. 75.0–120.0], INR 1,25 [ref. 0.80–1.20], APTT 35.9 s [ref. 25.0–36.0], TT 20 s [ref. 12.0–18.0]) (**B**) one hour after fibrinogen substitution (Fbg 0.57 g/L [ref. 1.80–4.20], PT 76% [ref. 75.0–120.0], INR 1.24 [ref. 0.80–1.20], APTT 35.0 s [ref. 25.0–36.0], TT 21 s [ref. 12.0–18.0]) (**C**) four hours after fibrinogen replacement therapy (Fbg 0.45 g/L [ref. 1.80–4.20], PT 72% [ref. 75.0–120.0], INR 1.31 [ref. 0.80–1.20], APTT 36.2 s [ref. 25.0–36.0], TT 25 s [ref. 12.0–18.0]) (**D**) twelve hours after fibrinogen replacement therapy (Fbg 0.34 g/L [ref. 1.80–4.20], PT 62% [ref. 75.0–120.0], INR 1,48 [ref. 0.80–1.20], APTT 35.1 s [ref. 25.0–36.0], TT 28.3 s [ref. 12.0–18.0]).

In the diagnosis of hereditary hypofibrinogenemia, an abnormal median CT and MCF by FIBTEM assay is present compared to those of a dysfibrinogenemia diagnosis, of which the parameters of FIBTEM assay are ambiguous (median values of MCF are often higher in comparison with patients with hypofibrinogenemia). ROTEM, especially MCF and FIBTEM tests, are useful, e.g., in the perioperative management of fibrinogen replacement therapy in patients with a-, hypo-, or dysfibrinogenemia [45,46]. However, their effectiveness in predicting the clinical phenotypes of these disorders are unambiguous and must be confirmed by larger prospective studies [44,47]. A series of thromboelastometric curves after administration of 23.5 mg/kg fibrinogen concentrate in a patient with congenital afibrinogenemia is presented in Figure 8.

## 6. Limitations of ROTEM

The main limitation of the ROTEM test is the fact that it does not reflect the proportion of the endothelial component of hemostasis, collagen, nor platelet adhesion, and therefore, it is not suitable for the diagnosis of von Willebrand disease. ROTEM is insensitive to bleeding disorders related to abnormalities of blood vessels, too. Further, it is not possible to assess whether there is a deficit of one or more coagulation factors. Therefore, the examination does not consider the effect of hypothermia on the hemostasis mechanism since the system operates at a temperature of 37 °C, and the result may also distort the patient’s lower hematocrit when a falsely larger amplitude of the thromboelastometry curve is observed. Thromboelastometry has proven to be a reliable and rapid tool in the management of bleeding but did not significantly reduce excessive bleeding events or massive bleeding [48]. The necessity of manual pipetting on the ROTEM device, which is still required, is another limitation. This fact can lead to variability in the results, especially when it comes to blood clotting times. In their study, Kuiper, G.J.A.J.M. et al. [49] point out some artifacts in CT results and the inter-observer variability. They found out that CT results were shorter in male compared to female volunteers, with manual pipetting being the most likely cause of the discrepancies in their results [49].

## 7. Conclusions

The main limitation of conventional coagulation tests (PT/INR, aPTT, fibrinogen activity, and platelet count) is a static view of clot formation. Moreover, the time to perform these tests is too long to guide clinical decisions. On the contrary, viscoelastic methods (tromboelastography and ROTEM) provide a rapid and targeted view of thrombus formation, its stability, strength, and fibrinolysis process, thus allowing for the specific treatment of patients with coagulopathy, including the added benefit of detecting hypercoagulability and severe hyperfibrinolysis. Targeted hemostatic therapy with coagulation factor concentrates and fibrinogen concentrate in an adequate dose as part of a bleeding management algorithm significantly reduces the number of transfusions with all types of allogeneic blood products. Thus, the patient’s prognosis can be ultimately negatively affected [50], and their massive administration is associated with high morbidity and mortality. Following the implementation of ROTEM-based bleeding algorithms, the off-label use of recombinant activated factor VII (rFVIIa) was significantly reduced in situations where other therapeutic options failed. These algorithms not only reduce costs, but are also significant for the prevention of thrombotic events [51].

Today, the majority of the evidence supports the use of viscoelastic testing in the setting of transfusion management, but the vision for the future is to seek the possibility of its use it in the perioperative management of patients with inherited bleeding disorders, such as IFD or hemophilia. Since there is absence of a vascular wall component [52,53,54], the most common inherited bleeding disorder, von Willebrand disease, remains undetected by standard ROTEM. However, the ROTEM test is still useful in the ROTEM-controlled substitution of FVIII/vWF as well as in the substitution of fibrinogen in patients with IFD. Due to the rarity of these diseases, there is a limited amount of the data. Thus, further studies are necessary to validate the importance of ROTEM-controlled fibrinogen replacement therapy.

## Figures and Tables

**Table 1:** Summary of possible examinations and necessary reagents on the ROTEM^®^ delta device [9].

Assay	Investigated Area	Used Reagent
INTEM	Intrinsic coagulation pathway FXII, FXI, FIX, FVIII, FX, FV, FII, FI, fibrin, platelets, fibrinolysis	partial thromboplastin, ellagic acid
EXTEM	Extrinsic coagulation pathway FVII, FX, FV, FII, FI, fibrin, platelets, fibrinolysis	recombinant tissue factor, phospholipids
FIBTEM	Contribution of fibrinogen to the clot formation after platelet inactivation	recalcification and platelet inhibitor cytochalasin D
APTEM	Inhibition of fibrinolysis, comparison to EXTEM can indicate/detect hyperfibrinolysis	recalcification and fibrinolysis inhibitor aprotinin/tranexamic acid
HEPTEM	Heparin inactivation in heparinized patients	recalcification, heparinase I *

Legend: F—factor. * heparinase I (neutralase I) is an enzyme that specifically degrades heparin by catalyzing the cleavage of the saccharide bonds found in the heparin molecule.

**Table 2:** Reference values for ROTEM^®^ delta in adults (modified by [2]).

Assay	CT (s)	CFT (s)	Angle α (°)	A10 (mm)	A20 (mm)	MCF (mm)	LI30 (%)	ML (%) in 1 h
EXTEM	38–79	34–159	63–83	43–65	50–71	50–72	94–100	<15
INTEM	100–240	30–110	70–83	44–66	50–71	50–72	94–100	<15
FIBTEM	-	-	-	7–23	8–24	9–25	-	-
APTEM	A better clot formation in APTEM compared to EXTEM indicates an in vitro effect of antifibrinolytics (aprotinin and tranexamic acid)							
HEPTEM	A better clot formation in HEPTEM compared to INTEM indicates the presence of heparin or heparin-like anticoagulants							

**Table 3:** Recommended dose of fibrinogen for a targeted increase in FIBTEM A10 (A5) amplitude [38].

Targeted Increase in FIBTEM A10 (A5) (mm)	Dose of Fibrinogen (mg/kg)	Fibrinogen Concentrate (mL/kg)
2	12.5	0.6 (1 g per 80 kg)
4	25.0	1.2 (2 g per 80 kg)
6	37.5	1.9 (3 g per 80 kg)
8	50.0	2.5 (4 g per 80 kg)
10	62.5	3.1 (5 g per 80 kg)
12	75.0	3.8 (6 g per 80 kg)

## Data Availability

All the data are available from the corresponding author (tomas.simurda@uniba.sk) upon reasonable request.
